# Antimicrobial and Antibiofilm Effects of Bithionol against *Mycobacterium abscessus*

**DOI:** 10.3390/antibiotics13060529

**Published:** 2024-06-05

**Authors:** Dan Cao, Xin Yuan, Xiuzhi Jiang, Tiantian Wu, Yanghui Xiang, Zhongkang Ji, Jiaying Liu, Xu Dong, Kefan Bi, Tone Tønjum, Kaijin Xu, Ying Zhang

**Affiliations:** 1 State Key Laboratory for Diagnosis and Treatment of Infectious Diseases, National Clinical Research Center for Infectious Diseases, The First Affiliated Hospital, Zhejiang University School of Medicine, Hangzhou 310003, China; 2 Department of Microbiology, University of Oslo, Oslo University Hospital, 0424 Oslo, Norway; 3 Jinan Microecological Biomedicine Shandong Laboratory, Jinan 250117, China

**Keywords:** antimicrobial activity, biofilm, bithionol, drug screening, *Mycobacterium abscessus*

## Abstract

*Mycobacterium abscessus* (*M. abscessus*) is a multidrug-resistant nontuberculous mycobacterium (NTM) that is responsible for a wide spectrum of infections in humans. The lack of effective bactericidal drugs and the formation of biofilm make its clinical treatment very difficult. The FDA-approved drug library containing 3048 marketed and pharmacopeial drugs or compounds was screened at 20 μM against *M. abscessus* type strain 19977 in 7H9 medium, and 62 hits with potential antimicrobial activity against *M. abscessus* were identified. Among them, bithionol, a clinically approved antiparasitic agent, showed excellent antibacterial activity and inhibited the growth of three different subtypes of *M. abscessus* from 0.625 μM to 2.5 μM. We confirmed the bactericidal activity of bithionol by the MBC/MIC ratio being ≤4 and the time–kill curve study and also electron microscopy study. Interestingly, it was found that at 128 μg/mL, bithionol could completely eliminate biofilms after 48h, demonstrating an outstanding antibiofilm capability compared to commonly used antibiotics. Additionally, bithionol could eliminate 99.9% of biofilm bacteria at 64 μg/mL, 99% at 32 μg/mL, and 90% at 16 μg/mL. Therefore, bithionol may be a potential candidate for the treatment of *M. abscessus* infections due to its significant antimicrobial and antibiofilm activities.

## 1. Introduction

*Mycobacterium abscessus* (*M. abscessus*) is one of the most common nontuberculous mycobacteria, which can lead to chronic pulmonary diseases, soft-tissue infections, and other infections [1]. It comprises three subspecies: subsp. *abscessus*, subsp. *massiliense*, and subsp. *bolletii* [2]. The *M. abscessus* complex exhibits two distinct colony variants characterized by the presence (smooth morphotype) or absence (rough morphotype) of cell wall glycopeptidolipids (GPLs). The rough morphotype of *M. abscessus* exhibits higher virulence, leading to more severe pulmonary inflammatory responses, prolonged treatment periods, and poorer lung function [3,4]. *M. abscessus* is known to be one of the most drug-resistant of all mycobacteria and can lead to intractable lung diseases requiring long-term therapy due to intricate inherent and acquired resistance of the bacteria, along with the limited bactericidal activity of commonly used drugs [5]. In addition, *M. abscessus* has been reported to form biofilm in vivo within the airways and lung cavities of patients [6,7] and the development of biofilm aggregates is important for *M. abscessus* to endure the presence of antimicrobial agents [8]. However, commonly used antibiotics for the clinical treatment of *M. abscessus* have poor antibiofilm activity [9]. There is currently no standard treatment regimen for macrolide-resistant *M. abscessus* lung disease, highlighting the urgent need for both IV and oral, well-tolerated, antibiofilm, and bactericidal drugs. FDA-approved drugs have undergone rigorous testing for safety and efficacy and have well-characterized pharmacokinetics and toxicity profiles, including information on absorption, distribution, metabolism, and excretion. This study focused on screening the FDA-approved drug/compound library followed by further evaluation of the promising drug candidate bithionol for activity against *M. abscessus*.

## 2. Results

### 2.1. Screening the FDA Drug Library for Activity against M. abscessus

Of the 3048 drugs tested, we initially found 62 hits with potential antimicrobial activity against *M. abscessus* 19977. To confirm the screening results, the minimum inhibitory concentrations (MICs) of these 62 drugs were further evaluated against *M. abscessus* 19977. As shown in Table 1, the MIC values for the active compounds ranged from 0.02 μM to 20 μM. The antineoplastic agents bleomycin (hydrochloride) and bleomycin (sulfate) demonstrated the best antibacterial activity at the MIC of 0.02 μM. Bedaquiline, closantel, and closantel (sodium) could efficiently suppress the growth of *M. abscessus* at a low MIC of 0.625 μM. The MIC value of Clarithromycin, sitafloxacin, and tigecycline against *M. abscessus* was 1.25 μM. The MIC value of bithionol, auranofin, and rifamycin against *M. abscessus* was 2.5 μM. Twelve agents including regorafenib, gatifloxacin, rifamycin S, rifabutin, omadacycline, etrombopag (olamine), eltrombopag, ivermectin, doramectin, oxyclozanide, chlorhexidine, and hypericin could inhibit the growth of *M. abscessus*, with an MIC of 5 μM. In addition, we found 22 agents at the MIC of 10 μM and 17 agents at the MIC of 20 μM could inhibit the growth of *M. abscessus*. Although many of these drugs were known to have antibacterial activity, 35 active “hits” have not been previously reported for activity against *M. abscessus*, and their names are highlighted in bold in Table 1.

### 2.2. Activity against M. abscessus Clinical Strains

Excluding compounds with cytotoxic properties, known antiseptics, and drugs that have been reported to be useful against *M. abscessus*, seven agents including bithionol, eltrombopag, ivermectin, otilonium, fusidic acid, voxilaprevir, and gossypol were further studied. We evaluated their activity against clinical isolates of different subspecies of the *M. abscessus* complex, including clarithromycin-resistant and clarithromycin-susceptible strains. Among them, bithionol, eltrombopag, ivermectin, otilonium, fusidic acid, voxilaprevir, and gossypol showed potent activity against clinical isolates of the *M. abscessus* complex. As shown in Table 2, bithionol showed the best antibacterial activity and inhibited the growth of the *M. abscessus* complex at concentrations that ranged from 0.625 μM to 2.5 μM. The MICs for eltrombopag varied from 1.25 μM to 10 μM. The MICs for fusidic acid and voxilaprevir varied from 2.5 μM to 20 μM and the MIC was 2.5 μM–10 μM for ivermectin, 10 μM for otilonium, and 10 μM–20 μM for gossypol.

### 2.3. MIC and MBC of Bithionol against M. abscessus in Different Media

Given the drugs currently used to treat *M. abscessus* in clinical practice lack bactericidal activity, the bactericidal efficacy of bithionol against *M. abscessus* type strain ATCC 19977 and four clarithromycin-resistant clinical isolates 49, 97, 2136, and 2338 was tested through MBC/MIC in 7H9 medium supplemented with 1% glucose and CAMHB medium. As shown in Table 3, bithionol showed inhibition activity against *M. abscessus* in CAMHB medium ranging from 0.5 to 2 μg/mL. The MIC value of *M. abscessus* in 7H9 medium ranged from 0.25 to 0.5 μg/mL. The MBCs in 7H9 medium and CAMHB medium were 0.5–1 µg/mL and 1–4 µg/mL, respectively. The MBC/MIC ranged from 2 to 4 in both media. Therefore, bithionol displayed bactericidal activity against both clinical strains and *M. abscessus* type strain in 7H9 medium supplemented with 1% glucose medium and CAMHB medium. Furthermore, the antimicrobial activity of bithionol against *M. abscessus* ATCC 19977 was tested by agar dilution assay and was found to have an MIC of 0.5 μg/mL.

### 2.4. Time–Kill Assay

**Figure 1:**
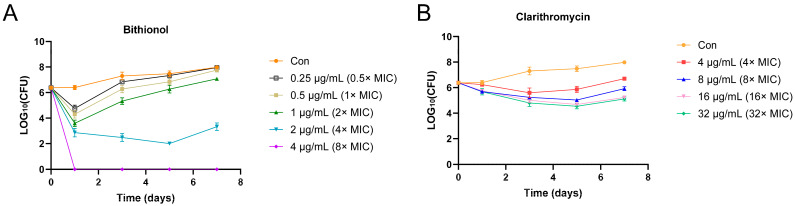
Time–kill curves of (**A**) bithionol and (**B**) clarithromycin against *M. abscessus* 19977 in 7H9 medium supplemented with 1% glucose at different drug concentrations. Drug concentrations are indicated by different symbols.

Further assessment of the bactericidal activity of bithionol was determined using a time–kill assay. The time–kill curve (Figure 1) indicated that bithionol, at a concentration of 4 μg/mL in 7H9 medium supplemented with 1% glucose, successfully eliminated all tested bacteria after 24 h. In addition, *M. abscessus* 19977 was reduced to about 10^3^ and 10^2^ in 24 h and 72 h at the concentration of 2 μg/mL (Figure 1A), respectively. However, clarithromycin did not exhibit bactericidal activity as a control drug (Figure 1B).

### 2.5. Antibiofilm Effect of Bithionol

**Figure 2:**
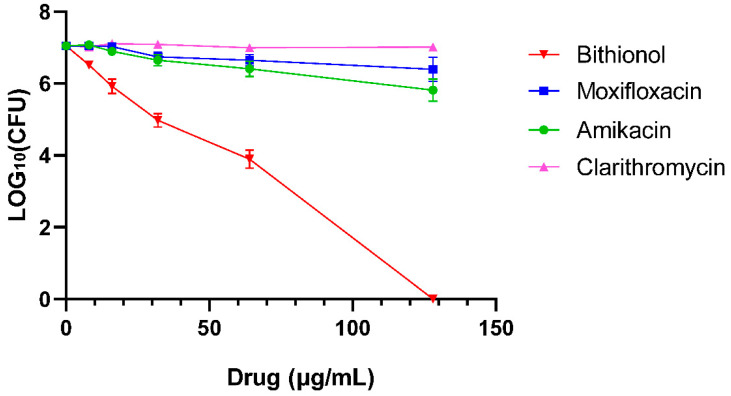
Determination of antibiofilm activity of bithionol. *M. abscessus* 19977 was cultured in Sauton’s medium in a 24-well plate at 37 °C for 6 days and biofilms formed on the liquid–air interface. Clarithromycin, amikacin, moxifloxacin, and bithionol at final concentrations of 8, 16, 32, 64, and 128 μg/mL were added to wells and incubated at 37 °C for 48h. Then, the biofilms were collected and CFU counts were determined.

As shown in Figure 2, the antimicrobial activities of clarithromycin, amikacin, and moxifloxicin as control drugs against *M. abscessus* biofilms were weak even at 128 μg/mL. However, bithionol exhibited a robust antibiofilm effect and could eradicate bacteria in the biofilm at 128 μg/mL. In addition, bithionol showed the ability to eliminate 99.9% of biofilm bacteria at 64 μg/mL, 99% at 32 μg/mL, and 90% at 16 μg/mL, respectively.

### 2.6. Scanning Electron Microscopy (SEM) Studies

**Figure 3:**
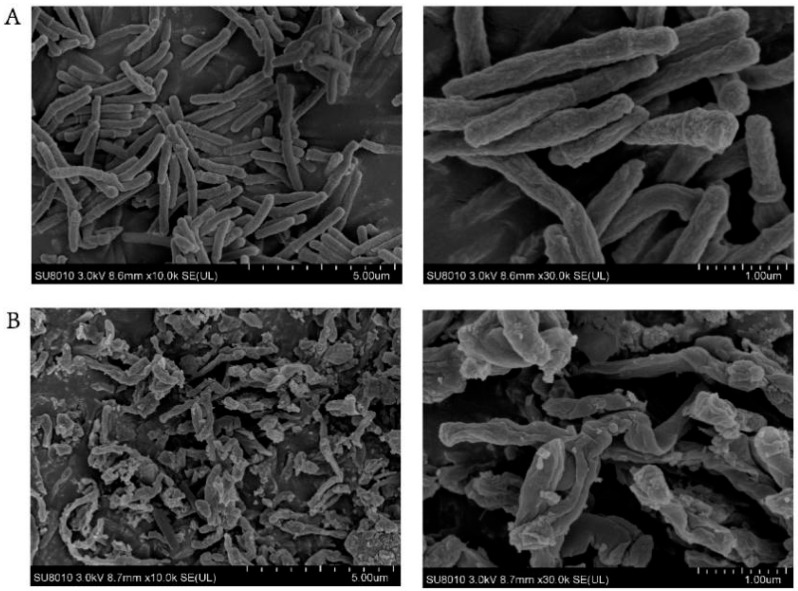
SEM images of bithionol treatment of *M. abscessus*. (**A**) *M. abscessus* control group treated with PBS; (**B**) *M. abscessus* bacteria treated with bithionol at 2 μg/mL for 24 h. The bars of the left panel indicate 5 μm, while the bars of the right panel indicate 1 μm.

To further investigate the mechanism of antibacterial action of bithionol, scanning electron microscopy (SEM) studies were carried out with *M. abscessus* type strain 19977 treated with bithionol at 2 μg/mL for 24 h. It was found that the bactericidal activity of the drug is related to its ability to cause abnormal cell morphology and cell lysis (Figure 3), resulting in the incomplete structure of the bacteria.

## 3. Discussion

*M. abscessus*, a member of the rapidly growing nontuberculous mycobacteria (NTM) [10], is particularly notorious for its resistance to multiple antibiotics, which poses a significant challenge for the treatment of *M. abscessus* infections [11]. Previous studies have found *M. abscessus* exhibits reduced susceptibility to several first-line antibiotics, including clarithromycin, cefoxitin, and amikacin, when grown in biofilm in vitro [12]. This underscores the critical and immediate need for the development of novel antibiotics specifically tailored to combat this bacterium, especially against biofilms. Although various chemical libraries have been used for high-throughput screening against *M. abscessus* [13,14,15,16,17,18,19,20], regrettably, there is a scarcity of active new drug candidates in both the clinical and discovery phases with varying results in different studies. In our study, the FDA Approved & Pharmacopeial Drug Library contained more chemicals, i.e., 3048 marketed and pharmacopeial drugs or compounds were utilized to identify new hits. Some of the useful agents screened in our study were also previously reported to be active against *M. abscessus* such as bedaquiline (fumarate), clarithromycin, amikacin (disulfate), sitafloxacin (hydrate), rifabutin, tedizolid, auranofin, omadacycline (hydrochloride), apramycin (sulfate), and ivacaftor [21,22,23,24,25,26,27,28]. Fortunately, we also discovered some potential drugs that have not been reported. After considering the cytotoxicity, seven commercial medications, including bithionol, eltrombopag, ivermectin, otilonium, fusidic acid, voxilaprevir, and gossypol, were further explored. We focused on evaluating bithionol in this study, and if conditions permit, other drug hits will also be further explored.

Bithionol is a clinically approved antiparasitic agent with proven safety in humans [29]. A recent study tested the cytotoxicity of bithionol on macrophage RAW 264.7 cells as well as HK-2 kidney cells using the CCK-8 method and found no significant cytotoxic difference between the effects of bithionol at 16 μg/mL and saline after treatment of 24 h and 72 h [30]. In a previous study, bithionol was found to effectively kill MRSA persister cells by disrupting the membrane integrity of Gram-positive bacteria [31]. In addition, bithionol showed significant antimicrobial and antibiofilm effects against *Enterococcus faecalis*, *Enterococcus faecium*, and *Staphylococcus aureus* [32,33]. Researchers found that bithionol showed synergistic antimicrobial and antibiofilm effects when used in combination with colistin against multidrug-resistant (MDR) organisms [30]. Recent research has revealed that bithionol inhibits PafA in an ATP-competitive manner and could suppress the growth of an attenuated *M. tuberculosis* strain H37Ra [34]. In our study, we assessed the minimum inhibitory concentration of bithionol against 10 clinical strains of *M. abscessus* and observed potent antibacterial efficacy against all 3 clinical strain types, with MIC values ranging from 0.625 to 2.5 μM. Since the drugs used for the treatment of *M. abscessus* lack bactericidal activity, we investigated the bactericidal efficacy of bithionol. A bactericidal antibiotic is one with an MBC to MIC ratio ≤ 4. Here, the MBC/MIC ratio ranged from 2 to 4 for the type strain and four clarithromycin-resistant isolates, indicating that bithionol is a bactericidal agent against *M. abscessus*. Furthermore, the time–kill assay demonstrated robust bactericidal efficacy, with complete eradication of *M.abscessus* after 24 h of treatment with bithionol at a concentration of 4 μg/mL. What is even more exciting is that bithionol, at a concentration of 128 μg/mL, could completely eradicate all biofilms after two days’ treatment. At 64 μg/mL, bithionol can eliminate 99.9% of biofilm bacteria. This efficacy remains strong at 32 μg/mL, where it eradicated 99% of biofilm bacteria, and at 16 μg/mL, is effective in killing 90% of biofilm bacteria. This demonstrates an excellent antibiofilm capability compared to commonly used antibiotics for *M. abscessus*. Subsequently, we conducted scanning electron microscopy and observed that the bactericidal mechanism of bithionol was associated with abnormal cell morphology and cell lysis, which may be related to its ability to achieve the sterilization of *M. abscessus*. In the future, we will further study the antibiofilm activity of bithionol using techniques such as microscopy.

Eltrombopag, a drug approved for the management of chronic idiopathic thrombocytopenic purpura, has previously been shown to be active against *Streptococcus pneumoniae*, multidrug-resistant *Staphylococcus aureus*, and *Staphylococcus epidermidis* [35,36]. In our study, eltrombopag was active against *M. abscessus* clinical isolates with an MIC between 1.25 μM and 10 μM. Ivermectin is an antiparasitic drug approved by the FDA, which is well tolerated and widely used for the treatment of onchocerciasis, helminthiasis, and scabies [37]. It has been shown that ivermectin has antimicrobial effects on a range of mycobacteria and also showed potent anti-staphylococcal activity [38]. In our study, ivermectin inhibited the growth of *M. abscessus* clinical isolates from 2.5 μM to 10 μM. A previous study found antispasmodic drug otilonium exhibited bactericidal activity against *Staphylococcus aureus* and could enhance the antibacterial activity of colistin against Gram-negative bacteria [39,40]. In this study, the MIC of otilonium (bromide) against *M. abscessus* clinical strains was 10 μM. Voxilaprevir is a direct-acting antiviral agent (DAA) used to treat chronic hepatitis C, and no antimicrobial effects have been reported [41]. Interestingly, we found the MIC of voxilaprevir against *M. abscessus* clinical strains ranged from 2.5 μM to 20 μM. Fusidic acid is an anti-staphylococcal antibiotic that has been used for the treatment of skin infections as well as chronic bone and joint infections [42]. There have been studies exploring the activity of fusidic acid against *M. tuberculosis* and it was found that it may be a therapeutic supplement for drug-resistant tuberculosis [43]. Here, we determined the susceptibility of fusidic acid against *M. abscessus* and found the MIC ranged from 2.5 μM to 20 μM. Gossypol is a natural product that exists in the seeds of cotton (Gossypium) and has been widely studied for cancer treatment, male contraception, and antiviral treatment [44]. Here, we found that gossypol was active against *M. abscessus* at 10–20 μM.

## 4. Materials and Methods

### 4.1. Bacterial Strains and Culture Conditions

*M. abscessus* ATCC 19977 type strain was purchased from the American Type Culture Collection (ATCC). Clinical isolates of *M. abscessus* subsp. *abscessus* (n = 6), subsp. *bolletii* (n = 2), and subsp. *massiliense* (n = 2) used in this study were acquired from the First Affiliated Hospital of Zhejiang University School of Medicine. Strains were grown at 37 °C in Middlebrook 7H9 broth (BD Difco) supplemented with 0.2% glycerol, 0.05% Tween-80, and 10% OADC enrichment. For drug susceptibility testing, bacteria were grown in Middlebrook 7H9 broth supplemented with 0.2% glycerol and 1% glucose.

### 4.2. Compounds and Drug Library

The FDA Approved & Pharmacopeial Drug Library procured from MedChemExpress (MCE) Inc. (Shanghai, China) contained 3048 marketed and pharmacopeial drugs or compounds approved by institutions such as the FDA, PMDA, NMPA, EMA, or pharmacopoeia including the United States Pharmacopoeia, Japanese Pharmacopoeia, British Pharmacopoeia, and other pharmacopoeia. A total of 2975 compounds were provided in 10 mM solution, 56 compounds were provided in 2 mM solution, and 17 compounds were provided in 3 mg/mL solution. The library was supplied in either DMSO or water and stored at −80 °C. Bithionol was purchased from MedChemExpress (MCE) Inc. (Shanghai, China) for further study. 

### 4.3. Screening Assay

Initially, 3031 drugs or compounds from the FDA drug library were screened at 20 μM and 17 drugs were screened at 6 μg/mL against *M. abscessus* ATCC 19977 to identify active compounds. Bacterial suspensions were diluted in 7H9 broth supplemented with 1% glucose to achieve a final inoculum of 5 × 10^5^ CFU/mL. Drugs that visually inhibited bacterial growth for 4 days at 37 °C were regarded as “hits”. Once the antimicrobial activity of the drugs was confirmed, the minimum inhibitory concentrations (MICs) were determined (see below).

### 4.4. Minimal Inhibitory Concentration Assay against M. abscessus

The active “hits” against *M. abscessus* type strain ATCC 19977 were serially diluted two-fold in 7H9 medium supplemented with 1% glucose in the range of 20 to 0.01 μM and inoculated in 96-well plates at 37 °C for 4 days. Ten clinical isolates of *M. abscessus* including 49, 97, 835, 905, 908, 1783, 1808, 2075, 2136, and 2338 were tested in 7H9 medium supplemented with 1% glucose for the MIC test of the candidate drugs. Moreover, the MICs of clarithromycin against *M. abscessus* 19977 and clinical isolates were determined from 16 μg/mL to 0.0312 μg/mL. In addition, the agar dilution method was used to determine the MIC of bithionol against *M. abscessus* ATCC 19977 on 7H11 agar plates containing 0.125, 0.25, 0.5, 1, 2, and 4 μg/mL of bithionol, as previously described [45]. The MIC was the lowest concentration that prevented visible growth.

### 4.5. Minimum Bactericidal Concentration against M. abscessus

Minimum bactericidal concentrations (MBCs) of bithionol were determined to distinguish bacteriostatic activity from bactericidal activity. *M. abscessus* ATCC 19977 and four clarithromycin-resistant *M. abscessus* clinical isolates were tested in CAMHB medium and 7H9 supplemented with 1% glucose. After being incubated for 4–5 days, wells with drug concentrations equal to or exceeding the MIC were checked. The MBC was the lowest concentration that killed 99.9% of the starting inoculum.

### 4.6. Time–Kill Curve

*M. abscessus* 19977 cultures were prepared at 2.5 × 10^6^ CFU/mL in 7H9 medium containing 1% glucose and exposed to clarithromycin (4, 8,16, 32 μg/mL) or bithionol (0.5, 1, 2, 4 μg/mL) at 37 °C without shaking. At 1, 3, 5, and 7 days, bacterial suspensions were collected by centrifugation, washed, and resuspended in PBS. After serial dilutions, the bacterial suspension was plated onto 7H11 plates for colony counting. The experiments were performed three times, and CFU data were presented by mean ± standard deviation.

### 4.7. Biofilm Assays

*M. abscessus* 19977 was resuspended in sterile PBS and diluted 100-fold in Sauton’s medium. This inoculum in 1.5 mL Sauton’s medium was grown stationary in a 24-well plate at 37 °C for 6 days and biofilms formed on the liquid–air interface. The liquid was aspirated with a syringe, then 3 mL clarithromycin, amikacin, moxifloxacin, and bithionol at final concentrations of 8, 16, 32, 64, and 128 μg/mL were added to wells and incubated at 37 °C for 48 h. PBS was added as a control. After 48 h, the biofilms were collected and resuspended in 1 mL PBS. After serial dilutions, the bacterial suspension was plated onto 7H11 plates for colony counting.

### 4.8. Scanning Electron Microscopy

For the scanning electron microscopy (SEM) study, *M. abscessus* 19977 was diluted to 1 × 10^6^ CFU/mL in 7H9 medium supplemented with 1% glucose and was treated with bithionol at 2 μg/mL for 24 h at 37 °C without shaking. Then, the bacteria were washed by PBS and fixed overnight with 2.5% *v*/*v* glutaraldehyde. The samples were treated with 30, 50, 70, 80, 90, 95, and 100% ethanol for 15 min and observed using a Cold Field Emission Scanning Electron Microscope at Zhejiang University.

## 5. Conclusions

We screened the FDA-approved drug library and identified 62 hits with significant antimicrobial activity against *M. abscessus* 19977. Among these hits, bithionol demonstrated outstanding in vitro efficacy against three distinct subspecies of *M. abscessus* and drug-resistant strains. Additionally, bithionol exhibited rapid bactericidal activity and strong antibiofilm properties. Further studies are needed to evaluate if bithionol has activity against *M. abscessus* in animal models and has the potential to treat *M. abscessus* infections in humans.

## Figures and Tables

**Table 1:** Active “hits” identified from the initial screening against *M. abscessus* 19977.

Product Name	CAS No.	M.Wt	Research Area	Clinical Information	MIC (μM)	MIC (μg/mL)
Bleomycin (hydrochloride)	67763-87-5	1451.00	Cancer	Launched	0.02	0.23
Bleomycin (sulfate)	9041-93-4	1512.62	Cancer	Launched	0.02	0.24
Bedaquiline (fumarate)	845533-86-0	671.58	Infection	Launched	0.625	0.42
**Closantel**	57808-65-8	663.07	Infection	No Development Reported	0.625	0.41
**Closantel (sodium)**	61438-64-0	685.06	Infection	No Development Reported	0.625	0.43
Clarithromycin	81103-11-9	747.95	Infection; Cancer	Launched	1.25	0.93
Sitafloxacin (hydrate)	163253-35-8	436.84	Infection	Launched	1.25	0.55
Tigecycline (tetramesylate)		970.07	Infection; Cancer	Launched	1.25	1.21
**Bithionol**	97-18-7	356.05	parasite	Launched	2.5	0.89
Auranofin	34031-32-8	680.50	Cancer; Infection; Inflammation/Immunology	Launched	2.5	1.70
Rifamycin (sodium)	14897-39-3	719.75	Infection	Launched	2.5	1.80
**Regorafenib (Hydrochloride)**	835621-07-3	519.28	Cancer	Launched	5	2.60
Gatifloxacin (hydrochloride)	121577-32-0	411.86	Infection	Launched	5	2.06
Rifamycin S	13553-79-2	695.75	Infection	No Development Reported	5	3.48
Omadacycline (hydrochloride)	1196800-39-1	593.11	Infection	Launched	5	2.97
**Eltrombopag (Olamine)**	496775-62-3	564.63	Cardiovascular Disease; Cancer	Launched	5	2.82
**Eltrombopag**	496775-61-2	442.47	Cardiovascular Disease; Cancer	Launched	5	2.21
**Ivermectin**	70288-86-7	875.09	Infection; Cancer	Launched	5	4.38
Rifabutin	72559-06-9	847.00	Infection	Launched	5	4.24
**Doramectin**	117704-25-3	899.11	Infection	No Development Reported	5	4.50
**Oxyclozanide**	2277-92-1	401.46	Infection	No Development Reported	5	2.01
Chlorhexidine	55-56-1	505.45	Infection	Launched	5	2.53
**Hypericin**	548-04-9	504.44	Cancer	Phase 1	5	2.52
**Lapatinib (ditosylate)**	388082-77-7	925.46	Cancer	Launched	10	9.25
**Entrectinib**	1108743-60-7	560.64	Cancer	Launched	10	5.61
**Ebselen**	60940-34-3	274.18	Cancer; Infection; Inflammation/Immunology; Neurological Disease	Phase 3	10	2.74
**Mirodenafil (dihydrochloride)**	862189-96-6	604.59	Others	Launched	10	6.05
Tedizolid	856866-72-3	370.34	Infection	Launched	10	3.70
**Olverembatinib (dimesylate)**	1421783-64-3	724.77	Cancer	Launched	10	7.25
**Olverembatinib**	1257628-77-5	532.56	Cancer	Launched	10	5.33
**Sonidegib**	956697-53-3	485.50	Cancer	Launched	10	4.86
**Sonidegib (diphosphate)**	1218778-77-8	681.49	Cancer	Launched	10	6.81
**Idarubicin (hydrochloride)**	57852-57-0	533.95	Cancer; Infection	Launched	10	5.34
Fidaxomicin	873857-62-6	1058.04	Infection	Launched	10	10.58
**Marbofloxacin**	115550-35-1	362.36	Infection	No Development Reported	10	3.62
Levofloxacin (hydrate)	138199-71-0	370.38	Infection	Launched	10	3.70
Ciprofloxacin (hydrochloride monohydrate)	86393-32-0	385.82	Infection	Launched	10	3.86
Roxithromycin	80214-83-1	837.05	Infection	Launched	10	8.37
**Otilonium (bromide)**	26095-59-0	563.57	Neurological Disease	Launched	10	5.64
**Tilmicosin**	108050-54-0	869.13	Infection	No Development Reported	10	8.69
**Benzethonium chloride**	121-54-0	448.08	Neurological Disease	Launched	10	4.48
Erythromycin Ethylsuccinate	1264-62-6	862.05	Infection	Launched	10	8.62
**Cetrimonium (bromide)**	57-09-0	364.45	Cancer	No Development Reported	10	3.64
Apramycin (sulfate)	65710-07-8	637.66	Infection; Cancer	Phase 1	10	6.38
**Aclacinomycin A hydrochloride**	75443-99-1	848.33	Cancer	Launched	10	8.48
**Gossypol**	303-45-7	518.55	Infection, Cancer	Launched	20	10.37
**Gossypol (acetic acid)**	12542-36-8	578.61	Cancer	Launched	20	11.57
**Abiraterone acetate**	154229-18-2	391.55	Cancer	Launched	20	7.83
**Fusidic acid**	6990-06-3	516.71	Infection	Launched	20	10.33
Rifaximin	80621-81-4	785.88	Infection	Launched	20	15.72
Ivacaftor	873054-44-5	392.49	Endocrinology	Launched	20	7.85
**Mitoxantrone**	65271-80-9	444.48	Cancer	Launched	20	8.89
Tamoxifen (Citrate)	54965-24-1	563.64	Cancer	Launched	20	11.27
**Voxilaprevir**	1535212-07-7	868.93	Infection	Launched	20	17.38
**Rafoxanide**	22662-39-1	626.01	Infection	No Development Reported	20	12.52
Crystal Violet	548-62-9	407.98	Infection	Phase 3	20	8.16
Novobiocin (Sodium)	1476-53-5	634.61	Infection; Cancer	Launched	20	12.69
**Zinc Pyrithione**	13463-41-7	317.69	Cardiovascular Disease	Launched	20	6.35
Nitroxoline	4008-48-4	190.16	Infection; Cancer	Launched	20	3.80
**Fusidic acid (sodium salt)**	751-94-0	538.69	Infection	Launched	20	10.77
**Cetylpyridinium (chloride monohydrate)**	6004-24-6	358.00	Infection	Launched	20	7.16
Amikacin (disulfate)	39831-55-5	781.76	Infection	Launched	20	15.64

**Table 2:** The MIC (μM) of active candidates against 10 *M. abscessus* clinical isolates.

Isolate	Subspecies	Morphotype	Clarithromycin Susceptibility	MIC (μM)						
				Bithionol	Eltrombopag	Ivermectin	Otilonium (Bromide)	Voxilaprevir	Fusidic Acid	Gossypol
49	*abscessuss*	Smooth	Resistant (≥16 μg/mL)	0.625	5	5	10	20	10	20
97	*abscessuss*	Smooth	Resistant (≥16 μg/mL)	1.25	5	5	10	20	20	20
2338	*abscessuss*	Rough	Resistant (≥16 μg/mL)	1.25	5	2.5	10	10	20	10
2136	*abscessuss*	Smooth	Resistant (≥16 μg/mL)	1.25	5	5	10	10	10	10
835	*abscessuss*	Smooth	Sensitive (2 μg/mL)	2.5	2.5	2.5	10	2.5	20	20
908	*abscessuss*	Rough	Sensitive (1 μg/mL)	1.25	2.5	10	10	20	2.5	20
905	*massilience*	Smooth	Sensitive (0.0625 μg/mL)	2.5	5	5	10	10	20	20
2075	*massilience*	Rough	Sensitive (0.0625 μg/mL)	1.25	1.25	5	10	20	2.5	20
1808	*bolletii*	Smooth	Sensitive (2 μg/mL)	2.5	5	2.5	10	10	20	10
1783	*bolletii*	Smooth	Sensitive (0.5 μg/mL)	2.5	10	2.5	10	10	20	20

**Table 3:** MIC and MBC of bithionol against type strain ATCC 19977 and clarithromycin-resistant clinical isolates of *M. abscessus*.

Isolate	MIC (7H9) μg/mL	MBC (7H9) μg/mL	MIC (CAMHB) μg/mL	MBC (CAMHB) μg/mL
49	0.25	1	0.5	2
97	0.25	0.5	0.5	2
2136	0.25	0.5	0.5	2
2338	0.25	0.5	0.5	1
19977	0.5	1	2	4

## Data Availability

Data will be made available upon reasonable request to the authors.
